# Going Beyond the Data as the Patching (Sheaving) of Local Knowledge

**DOI:** 10.3389/fpsyg.2018.01926

**Published:** 2018-10-09

**Authors:** Steven Phillips

**Affiliations:** Mathematical Neuroinformatics Group, Human Informatics Research Institute, National Institute of Advanced Industrial Science and Technology, Tsukuba, Japan

**Keywords:** learning, generalization, sheaf theory, sheaf, sheaving, category theory, universal

## Abstract

Consistently predicting outcomes in novel situations is colloquially called “going beyond the data,” or “generalization.” Going beyond the data features in spatial and non-spatial cognition, raising the question of whether such features have a common basis—a kind of systematicity of generalization. Here, we conceptualize this ability as the patching of local knowledge to obtain non-local (global) information. Tracking the passage from local to global properties is the purview of sheaf theory, a branch of mathematics at the nexus of algebra and geometry/topology. Two cognitive domains are examined: (1) learning cue-target patterns that conform to an underlying algebraic rule, and (2) visual attention requiring the integration of space-based feature maps. In both cases, going beyond the data is obtained from a (universal) sheaf theory construction called “sheaving,” i.e., the “patching” of local data attached to a topological space to obtain a representation considered as a globally coherent cognitive map. These results are discussed in the context of a previous (category theory) explanation for systematicity, vis-a-vis, categorical universal constructions, along with other cognitive domains where going beyond the data is apparent. Analogous to higher-order function (i.e., a function that takes/returns a function), going beyond the data as a higher-order systematicity property is explained by sheaving, a higher-order (categorical) universal construction.

## 1. Introduction

A ubiquitous cognitive ability is the capacity to “go beyond the data.” That is, to put it broadly, an ability to successfully respond to stimuli not previously encountered. Such a characterization encompasses a wide variety of situations from perception-based classification to logic-like reasoning. For example, given feedback on the edibility of a particular kind of fruit, one knows when the fruit can be eaten next time it comes into season. Or, having been (repeatedly) rewarded for choosing stimulus *A* over stimulus *B* and *B* over stimulus *C*, one correctly predicts that choosing *A* over *C* will also elicit a reward. In general, a capacity to go beyond the data is referred to as *generalization*. And, this ability is typically expressed as correct responses to novel inputs given some knowledge about other input-output (cue-target) examples.

This broad view of generalization affords an instructive comparison/contrast of two distinctive views of cognition, to wit, *classical* (symbolic) and *connectionist* (subsymbolic/vectorial). The relative merits of these two views (Fodor and Pylyshyn, 1988) have been extensively debated in the literature (see Calvo and Symons, 2014, for a cross-section of arguments). Our interest, here, is with several key aspects of the debate that motivate and help illustrate a different conception of generalization to follow.

The classical view is that our ability to reason about the world is founded upon a compositional syntax and semantics: the world is interpreted through a *language of thought* (Fodor, 1975). A language of thought is a system of representations—complex entities are modeled by corresponding compositional representations so that the semantic relationships between the constituent entities are reflected in the syntactic relationships between the corresponding constituent representations—and processes that are compatible with the way such compositional representations are constructed. So, for instance, on seeing that John is standing to the left of Mary, there is a symbol representing John juxtaposed with a symbol representing Mary in a way that captures the relative spatial locations of John and Mary. What matters to the classical theory is not the particular syntactic relationship, but that the relationship employed is used consistently in all such situations. In this way, a classical cognitive system with the capacity to juxtapose all such relevant combinations of symbols is supposed to explain the *productivity* and *systematicity* properties of language (Chomsky, 1980) and thought (Fodor and Pylyshyn, 1988), more generally.

Productivity and systematicity can be seen as forms of generalization in the sense just introduced. Productivity, as the term suggests, is characterized as having a cognitive capacity that is more than the sum of its parts. For instance, suppose in the course of understanding the meaning of “to the left of” that upon being told, “John is standing to the left of Mary,” “Mary is standing to the left of Tom,” and “John is standing to the left of Tom” when indeed one sees that John is to the left of Mary, and so on, that symbols are recruited to represent John, Mary and Tom. Suppose, further, that a (product) rule is exercised to combine that set of symbols into a set of symbol pairs: e.g., (John, Mary) in corresponding (left, right) order, together with a process for accessing the first symbol in each pair, thereby affording the inference that John is the person on the left when applied to the pair (John, Mary). This system exhibits productivity, therefore generalization in the aforementioned sense, because a basic capacity to represent three pairs of people produces (generalizes to) a capacity to represent all six possible pairs of people without further instruction. A similar consideration applies to systematicity: where having the capacity for one such instance implies having the capacity for another (structurally-related) instance, via application of the same combinatorial process (Fodor and Pylyshyn, 1988; Aizawa, 2003).

A connectionist view, which eschews symbolic representations and processes, is that our ability to reason about the world is founded upon vector (coordinate) based representations and processes, realized as networks of neuron-inspired computational units (Rumelhart et al., 1986). A connectionist model employs vectorial representations—complex entities are modeled by corresponding vectors so that the semantic relationships between the constituent entities are reflected in the spatial (geometrical) relationships between the corresponding constituent representations—and functions that are compatible with the way such vectorial representations are constructed. In the linear case, where the computational units involve only linear functions, connectionist models can provide analogous accounts of productivity and systematicity via linear algebra (Smolensky, 1990). In the non-linear case, where units involve non-linear functions, productivity and systematicity, and other forms of generalization, obtain from judicious choices of learning methods and non-linear functions (see e.g., Hadley, 1994; Frank et al., 2009, among many others).

Although classical and connectionist approaches can demonstrate various generalization properties, they both fall short of an important theoretical challenge. That challenge is to explain *why*, not just how properties such as systematicity derive from the core principles of the theory. This challenge was the one originally raised against connectionist theories (Fodor and Pylyshyn, 1988), and later shown to be problematic for classical theories too (Aizawa, 2003). The essence of the problem is that the core principles admit systems that do *and* systems that do not exhibit systematicity. In both cases, the core theoretical claims do not derive the systematicity properties without tailoring auxiliary assumptions to fit the data whenever such properties are evident. Such assumptions are characteristically *ad hoc* in being unconnected to the core principles of the theory, motivated solely to fit the data, and cannot be confirmed independently of confirming the theory—accordingly, classical and connectionist theories fail to *fully* explain such properties (Aizawa, 2003).

To address this challenge, a *category theory* (Eilenberg and Mac Lane, 1945; Mac Lane, 1998) approach was proposed whereby systematicity properties derive from *universal (categorical) constructions* (Phillips and Wilson, 2010). For example, of the many possible ways of combining symbols or vectors to represent pairs there is only “one” way (see remark 8 in Appendix of the Supplementary Material) to combine them so that the constituents are uniquely accessible in every possible case, called the *categorical product*. Various scenarios for systematicity were explained in terms of appropriate universal constructions (see Phillips and Wilson, 2016b, for an overview). A summary of the systematicity challenge, which motivates the categorical theory approach is given in the last section of the Appendix (Supplementary Material).

An explanation for systematicity, however, raises to a wider question, Why do people fail to exhibit systematicity in some situations? In particular, failure to apply certain rules of inference (*modus ponens* and *modus tollens*) in the relevant situations at least calls into question the classical account of systematicity (van Gelder and Niklasson, 1994). Other forms of fallacious reasoning, such as the *conjunction/disjunction fallacy* (Tversky and Kahneman, 1983) and *pseudo-transitive inference* (Goodwin and Johnson-Laird, 2008), raise a similar challenge. A general framework within which such questions and challenges may be addressed is called *dual-process* (see Evans, 2003, for a review).

Dual-process accounts of cognition assume two modes of thinking, generically labeled *Type 1* and *Type 2*, which are typically characterized as fast, reflexive, associative and relatively effortless—Type 1—vs. slow, reflective, rule-based and relatively effortful—Type 2 (Kahneman, 2011; Evans and Stanovich, 2013). The basic idea is that the two systems trade off complementary properties so that, for example, under time pressure a faster Type 1 process may supersede a slower Type 2 process yielding an incorrect response (Kahneman, 2011; Evans and Stanovich, 2013)—the two types of processes trade the benefit of speed for the cost of accuracy.

Along similar lines, a trade-off was hypothesized in regard to systematicity: for the categorical account, lack of systematicity is due to the relative cost/benefit of constructing the appropriate universal morphism (Phillips et al., 2016). This hypothesis was tested in a stimulus-response learning experiment, where the maps to be learned were *products* of cue-target maps. The supposed trade-off involved learning a single (associative) route of *n*^2^ mappings vs. a pair of routes (via a product-rule) of 2*n* mappings—increased memory vs. decreased attention. Two groups of participants were administered the task. The *ascend* group were trained and tested on four different cue-target maps in ascending order of map size (i.e., from three-by-three to six-by-six possible cue-target associations). This group showed generalization (correct responses) to novel stimuli in the testing set only when the number of cue-target pairs to be learned was large, indicating that they did not construct the universal morphism (product map) for small maps, even though there were sufficient training examples to induce the construction. The *descend* group were trained and tested in descending order of map size. This group showed generalization to the testing set at all sizes, indicating systematic induction of the product map. Together, these results support a cost/benefit explanation (Phillips et al., 2016).

The cost/benefit explanation, as it pertains to the experiment, raises two closely related questions: (1) what determines the choice of (associative vs. [product] rule-based) learning route, and (2) in the case of the rule-based learning route, how/why are universal morphisms systematically constructed? This paper is primarily concerned with the second question: under the assumption that participants are driven toward the rule-based route, how/why are universal morphisms constructed? We return to the broader question of how/why participants are driven to this learning route, i.e., the interaction between cost/benefit and the construction of universal morphisms in the Discussion. To the second question, then, the consistent (systematic) transition from no systematicity (no universal construction) to systematicity (universal construction) itself suggests another form of universal construction. These considerations, which constitute the starting point for the current work, lead naturally to another (closely related) branch of mathematics, called *sheaf theory* (Hartshorne, 1977; Mac Lane and Moerdijk, 1992), applied here as a basis for generalization.

The import of sheaf theory to cognitive science may seem obscure. So, a preview of the sheaf theory approach is provided in the remainder of this introduction before delving deeper into the conceptual details and cognitive applications (subsequent main text), and supporting formal theory (Appendix in Supplementary Material).

### 1.1. Preview: generalization as patching (Sheaving)

A capacity to generalize beyond the given instances connotes a property that is (re)constructed from local information. Conceptually, at least, this situation is akin to tracking the passage from a local to a global property, which is the purview of sheaf theory. This way of looking at generalization renders the essential ingredients (axioms) of sheaf theory as a formalization of some classical and connectionist concepts already introduced. In this light, the path from sheaf theory to cognition is less abstruse. Indeed, sheaf theory is where algebra meets geometry/topology. If one regards classical and connectionist approaches as complementary, which some researchers do (e.g., Holyoak and Hummel, 2000; Clark et al., 2008)—a language of thought (Fodor and Pylyshyn, 1988) on one hand and a geometry of thought (Gardenfors, 2000) on the other, then sheaf theory alludes to a natural integration of the two.

There are three fundamental aspects to sheaf theory that we will interpret in terms of cognitive representations and processes: (1) *presheaf* (*sheaf*), a basic element of sheaf theory, which we will regard as a (coherent) cognitive map or representation, (2) *sheaving*, the (universal) process of constructing a sheaf from a presheaf, which we will interpret as a form of systematic generalization, and (3) *sheaf morphism*, regarded here as a kind of inference, i.e., a cognitive process acting on a cognitive representation.

A sheaf is like a work of art, and sheaf operations are like the artistic process, in the following sense. To create a portrait, an artist applies paint to canvas. The canvas is a topological space and the paint is the data attached to that space. As a work in progress, there are unpainted regions on the canvas, or sections of the portrait that don't quite match. In unfinished form, the portrait is a presheaf. Paint is added to the vacant regions, or laid over existing sections to obtain the finished form. This patching process is likened to sheaving, and the finished form to a sheaf. The finished portrait may be further altered, e.g., by changing tone to affect mood, thus creating a new portrait, and this process is likened to a sheaf morphism.

This artistic rendering of sheaves has analogs in classical and connectionist theory. The canvas is a representational space in which symbolic, or vectorial representations are constructed, combined (patched), or transformed. As we shall see, sheaf theory provides a formal basis for such processes and, in particular, generalization as the patching of local knowledge to obtain non-local (global) information. We present the basic sheaf theory and the sheaving construction considered as a “universal” basis for generalization (section 2). Then we examine sheaving in two cognitive domains (section 3): cue-target learning, involving the product of two cue-target maps, and visual search involving the integration of two visuospatial maps. In terms of sheaves, the first domain is a special case of the second domain. These results are discussed in the context of a previous (category theory) explanation for systematicity, vis-a-vis, categorical universal constructions, along with other cognitive domains where going beyond the data is apparent (section 4). Supporting technical material is provided in the Appendix (Supplementary Material).

## 2. Sheaves and sheaving

As previewed in the Introduction, sheaf theory concerns the passage from local to global properties, which we interpret as generalization in the context of cognition. This section provides a conceptualization of the formal details. Although the presentation in this section is primarily intuitive, some notation is included to facilitate links to the formal theory. Sheaf theory is initially given in terms of sets and functions. However, a category theory view is particularly relevant here, because of our interest in universal constructions as an explanatory basis for systematicity (generalization). In short, there are two levels of universality: one at the level of sheaf, which is defined via a universal construction, and the other at the level of collection of sheaves that pertains to sheaving, which is another kind of universal construction. Accordingly, sheaving pertains to a kind of second-order systematicity (see Chomsky, 1980; Aizawa, 2003; Phillips and Wilson, 2016a), alluded to earlier. Moreover, a category theory approach affords wider applicability to domains involving more structure than just sets. A guide to formal concepts for the various theoretical views and their relationships is given in Table 1.

**Table 1:** Corresponding set/relational database and category/sheaf theory concepts.

**Set theory**	**Relational database theory**
Element, set	Column name, header
(assignment) function	(data) table
(higher-order) function	(table) transformation
optimal function	natural join, renormalization
**Category theory**	**Sheaf theory**
Object/morphism, category	Open set/inclusion, topology
(contravariant) functor	presheaf/sheaf
natural transformation	presheaf/sheaf morphism
universal morphism	pullback, sheaving

Conceptually, one can think of sheaving as a process of obtaining a coherent “map” or representation of a complex situation that is a sheaf. Hence, the state of affairs *before* having a sheaf is called a *pre*sheaf. Sheaving is a (universal) way of going from a presheaf to a sheaf. An immediately intuitive example is navigating a city using a street directory. Each page of the directory contains a map of a local area. To visit a distant part of town, pages mapping contiguous areas must be “glued” together along common landmarks to yield a map that includes both the current location and the destination. Gluing all such pages is akin to constructing a sheaf, and the construction process is akin to sheaving.

The street directory example is intended to bootstrap some basic intuitions about presheaves and sheaves. A presheaf, or a sheaf is an assignment of data (sets of elements) to (regions of) a topological space, where a sheaf is required to satisfy some additional coherence conditions. In the context of the street directory example, we have the following interpretation.

- A *topological space* is a set of elements together with a collection of its subsets, which indicates the relative proximity of those elements (definition 6 in Appendix of the Supplementary Material). The collection (set) of subsets is called the *topology* of the space, and the subsets are the *open sets* of the topology. So, the pages of the street directory correspond to open sets.
- A *presheaf* is an assignment of data to the open sets of the topological space. The assignment is given by a function that sends each open set to some set (definition 16 in Appendix of the Supplementary Material). The data in the current example are the markings that constitute the street map *on* each page of the directory. The assignment of data to the open sets is required to satisfy a certain *restriction* condition, which says that the data assigned to an open set *V*⊆*U* is the data assigned to open set *U* restricted to *V* (definition 4 in Appendix of the Supplementary Material). For instance, this condition says that the markings on two adjacent pages treated as a single page restrict to the markings on the individual pages. For a presheaf, in general, the restrictions of data to the intersection of open sets need not agree. This situation occurs when, for example, the map for an urban area may contain more detailed information than the map for an adjacent nature reserve so that the two sets of landmarks shown for the overlapping area are not the same set.
- A *sheaf* is a presheaf such that the data for overlapping open sets is the same (definition 17 in Appendix of the Supplementary Material). There are two conditions for a presheaf to be a sheaf (definition 17 in Appendix of the Supplementary Material). The requirement that the data agree on overlaps is called the *gluing* condition; the requirement that the gluing be unique is called the *locality* condition, which essentially says that there is no ambiguity in the way local information is patched together.
- *Sheaving* (definition 18 in Appendix of the Supplementary Material) is a universal way (theorem 1 in Appendix of the Supplementary Material) of constructing the “best” possible sheaf from a given presheaf (example 9 in Appendix of the Supplementary Material).

The foregoing illustration, though intuitive, glosses over important details that may leave one questioning the motivation for a sheaf theory approach. Firstly, why are we concerned with the more abstract notion of topological space, rather than the more concrete notion of coordinate space used, for example, in connectionist models where the vectors take on real numbers? Secondly, how are data attached to a topological space? Thirdly, in what sense does sheaving return the “best” possible sheaf for a given presheaf? Each question is addressed in the next three sections, in turn.

### 2.1. A topological view of space

The street map example may leave one wondering about the need to work with a topological space. A topological approach is appropriate when there is no suitable notion of “distance,” between representations, as required by a *metric space*. The extra abstraction also affords a parsimonious treatment of symbolic and numeric (coordinate) representations, as both sets of symbols and sets of numbers can be given a topology.

To illustrate how symbols have a (topological) order without defining a distance measure, suppose we have a set of abstract symbols, *X* = {*A, B, C*}. A topology on *X* is a collection *T* of subsets of *X* that, at least, includes the empty set, ∅, and *X*. These subsets are designated as the *open sets* of *T* and indicate the relative proximity of the elements in *X*. For example, suppose *T* = {∅, {*C*}, {*B, C*}, {*A, B, C*}}. This topology is a *specialization order topology* corresponding to the order *A* ≤ *B* ≤ *C*, which says that *B* is closer to *C* than *A*.

Every set *X* can be given two extreme topologies (example 1 in Appendix of the Supplementary Material), which have associated orders. One extreme is called the *indiscrete topology*, which contains just the empty set and *X*. The other extreme is called the *discrete topology*, which contains every subset of *X*. The (*pre*)order associated with the indiscrete topology on {*A, B*} has the order relations *A* ≤ *B* and *B* ≤ *A*, and the order associated with the discrete topology has just the order relations *A* ≤ *A* and *B* ≤ *B*. Both can be interpreted as reflecting “minimal” information about the proximity of *A* and *B*.

Open sets are fundamental to the topological notion of space and continuity. Not only do they indicate proximity, but also how the regions of space relate to each other via inclusion. If two regions are open sets, then their intersection and union are also open sets (regions) of that space. As we shall see, next, intersections pertain to gluing and unions pertain to coverage. We have already introduced the importance of gluing, the importance of coverage is similarly efficacious—we require as many pages as needed to cover all city regions. Likewise, a representational space should cover the things that need to be represented. The corresponding concept in topology is *open cover* (definition 8 in Appendix of the Supplementary Material).

Perhaps less obvious is the role of opens sets to the property of being continuous. A function between topological spaces is called *continuous* if the *preimage* of an open set is an open set (definition 7 in Appendix of the Supplementary Material)—a continuous transformation obtains closely related things from closely related things. Notice that this topological definition admits continuous functions over “discrete” symbols. Being continuous is a property of a function, not a space. Connectionist representations are sometimes regards as “continuous” and symbolic representations as “discrete.” However, this difference is more akin to the difference between countable vs. uncountable sets: e.g., the set of natural numbers vs. the set of real numbers.

### 2.2. A relational view of data

The foregoing conception of representational space lays the groundwork, as it were, for a parsimonious treatment of representation as data attached to a (topological) space. A crucial observation is that relational databases can be viewed as presheaves, or sheaves (Abramsky and Brandenburger, 2011) (Note that presheave/sheaves are more general constructions than relational tables, because every point/column, or combination of points/columns need not constitute an open set of the topology). A relational database table consists of a header, listing the names of each column of a table, and rows that contain the data for that table. A collection of tables constitutes a relational database, and the headers constitute the relational schema. In terms of sheaf theory, the schema corresponds to a topological space, where each header is an open set, and the rows of a table correspond to the data attached to an open set. Thus, a relational database corresponds to a presheaf, or a sheaf when the tables can be glued together to give another table that is also part of the database. The relational database operation that realizes gluing is called the *natural join* (example 6 in Appendix of the Supplementary Material). A natural join, described in detail next, combines two tables into a single table whose rows are just those constructed by combining (joining) the rows from each table that have the same value at the columns in common. When there are no columns with the same name, i.e., the intersection of the table headers is the empty set, the natural join reduces to the (Cartesian) product of the two tables. The next section illustrates this situation first, and the section that follows illustrates the case where the two tables have common columns. For reasons that will become clearer later, the second situation is called a *constrained product*. From a relational database view, sheaving involves constructing tables not already in the database by gluing together existing tables. This process is also described next, and applied to cognition in section 3.

#### 2.2.1. Gluing as a product

**Figure 1:**
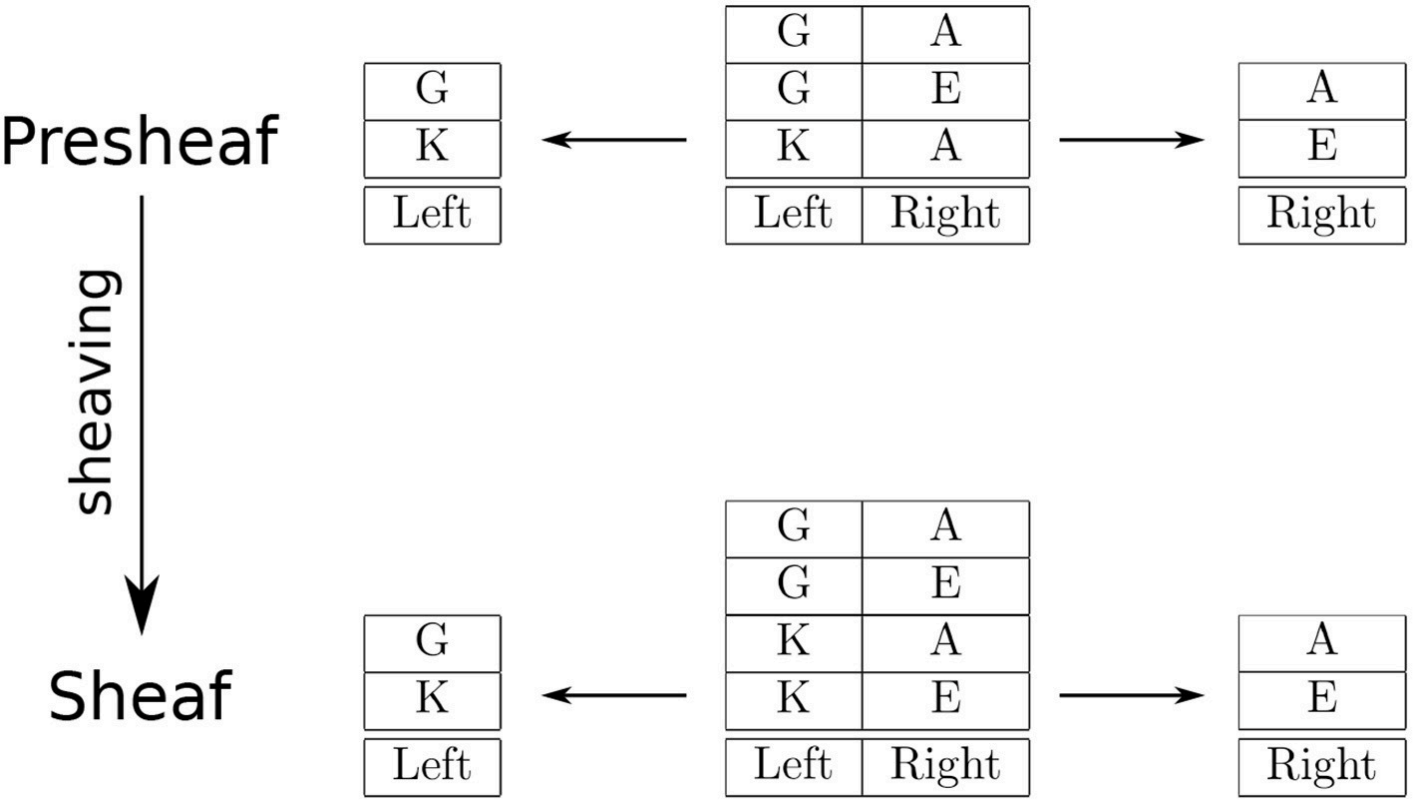
An example of sheaving as a product.

To illustrate gluing as a product, suppose we are given a pair of objects (characters/letters) in the visual field of view. The knowledge that the pair of characters (G, A) differs from the pair (A, G) is captured by recognizing the relative locations of each object: e.g., the first character is located at the left position, and the second character is located at the right position. Suppose the pairs (G, E) and (K, A) were also presented on separate occasions. This information is recorded in a relational database table that has two columns, named Left and Right, and three rows containing the three pairs of characters at the corresponding positions. Each row corresponds to a character pair. In addition, there is a one-column table headed Left and a one-column table headed Right, recording the locations of each character individually. The location names and headers constitue a (discrete) topological space: (Location, Proximity), where Location = {Left, Right} is the set of locations, and Proximity = {∅, {Left}, {Right}, {Left, Right}} is the discrete topology on that set. This situation corresponds to a presheaf, where the rows are the data attached to the topological space, see Figure 1 (top row). (The data attached to the empty set is the singleton set {*}, i.e., the one-element set containing an element whose name is unimportant. The corresponding table, not shown, is the table with the empty header and one row containing the unnamed element.) For instance, the pair (G, A) appears as a row of the two-column table, and the individual characters as rows of the corresponding one-column tables. From a sheaf theory perspective, the topological space provides the ground on which the data are attached. Accordingly, the tables are shown with the “header” at the foot of the table—footer.

Relational databases come equipped with operations for extracting information from tables. One basic operation is called *projection*, which returns all the values at the named columns for all the rows of the specified table. For instance, the characters located at the left position are obtained by a projection onto the Left column of the two-column table (Figure 1, upper left arrow), and likewise for the characters located at the right position (Figure 1, upper right arrow). In sheaf theory terms, these projections are given by restrictions for the presheaf. Recall that a presheaf is an assignment of open sets to sets of elements that preserves inclusions as restrictions: for each inclusion in the underlying topological space there is a corresponding restriction map. So, in this example, the restriction corresponding to the inclusion {Left}⊆{Left, Right} is the projection onto the Left column; likewise, the restriction corresponding to {Right}⊆{Left, Right} is the projection onto the Right column (The empty set is included in every open set, so the corresponding restrictions send every row to the only element in the singleton set).

This relational database specifies a presheaf, but not a sheaf, because the two-column table cannot be (re)constructed as the gluing (product) of the one-column tables. Specifically, the product of the one-column tables results in the row that is the pair (K, E), which is not contained in the two-column table. To be a sheaf, the gluing condition essentially says that there must be a row in the two-column table that contains (restricts to) a given pair of rows from the one-column tables, which is not the case for the pair (K, E). Thus, this presheaf is not a sheaf.

The sheaving process turns a presheaf into a sheaf by gluing along overlapping regions. In terms of relational database operators, gluing is the natural join. When the overlap is the empty set, gluing is essentially the product of tables. In this example, the product is all pairwise combinations of rows from the one-column tables. Hence, sheaving adds the (K, E) pair to the two-column table. Thus, the updated relational database corresponds to a sheaf, see Figure 1 (bottom row). The sheaving construction is a map from the top row to the bottom row (Figure 1, left vertical arrow).

#### 2.2.2. Gluing as a constrained product

The essential difference between gluing as a product and gluing as a constrained product is that the intersection of the underlying open sets, to which the data are attached, is not the empty set. This situation often occurs in relational databases, e.g., where personal information about employees is stored in one table and work-related information in another table, and taking the natural join on the common employee-identifier column links the two kinds of information.

Visual cognition can be considered analogously where object features (e.g., location, color, or shape) are stored in separate tables that can be joined to recover information about feature conjunctions to identify objects, e.g., that the displayed objects are red square and green triangle, not red triangle and green square. For this situation, suppose that objects are indexed by location, and color and shape information are recorded in separate two-column tables with headers (Location, Color) and (Location, Shape), respectively. Here, we have a set of feature dimensions, Feature = {Location, Colour, Shape}, and a topology, Bind = {∅, {Location}, {Location, Colour}, {Location, Shape}, Feature}. This topological space associates color and shape more closely to location than each other, which is interpreted as saying that color and shape feature maps are more basic than color-shape conjunction maps.

**Figure 2:**
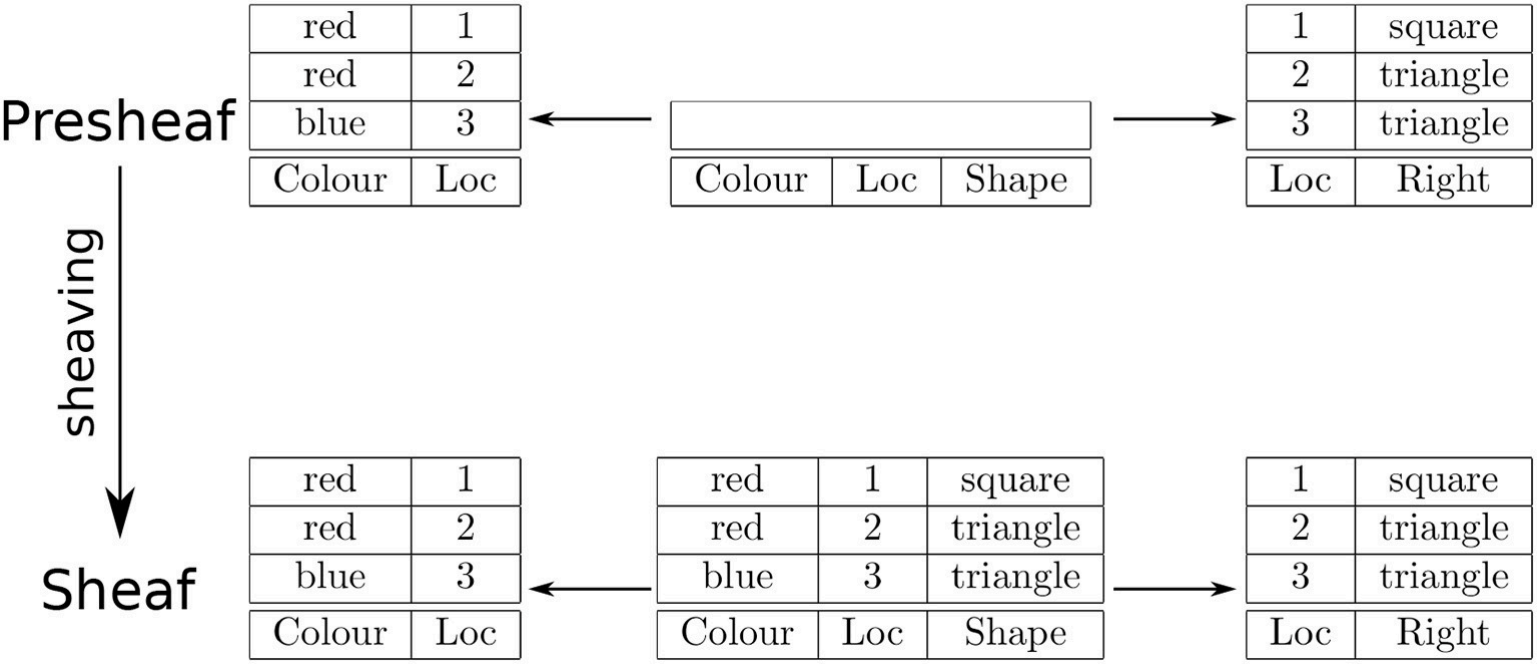
An example of sheaving as a constrained product (empty box indicates empty set).

In this situation, sheaving recovers the binding of color and shape as the natural join of Location-Color and Location-Shape tables, which results in the Location-Color-Shape table corresponding to a color-shape conjunction map. A psychologically compatible interpretation of this situation is binding-by-location (Treisman, 1996). An example is shown in Figure 2. The natural join in this case is constrained to return only those rows that agree on location, not all combinations of rows, as in the previous example. Hence, this case is called a constrained product.

Note that for the presheaf shown in Figure 2, the empty box indicates that the data attached to the open set is the empty set. Hence, all restrictions from this set are empty maps.

### 2.3. A category theory view of sheaves and sheaving (universality/systematicity)

Up to this point, we have presented the basic ideas of sheaves and sheaving in terms of sets and functions. This approach is easier to grasp, but obscures the importance of universal construction and its role in an explanation for systematicity and productivity. So, in this section, we present the category theory view of sheaves. The core concept that links sheaves, systematicity and generalization is *universal morphism* (definition 15 in Appendix of the Supplementary Material). This concept depends on the concepts of *category* (definition 9 in Appendix of the Supplementary Material) and *functor* (definition 13 in Appendix of the Supplementary Material), and is closely related to the concept of *natural transformation* (definition 14 in Appendix of the Supplementary Material). For a quick intuition, one can think of a category as a set with relations (*morphisms*) between its elements (*objects*), a functor as a function between categories that “preserves” those relations, and a natural transformation as a kind of higher-order function (i.e., a function that takes/returns a function, see remark 5 in Appendix of the Supplementary Material).

In the context of category theory, a topological space is a category with open sets for objects and inclusions for morphisms (example 2 in Appendix of the Supplementary Material). A presheaf (hence, a sheaf) is a functor from a topological space to the category set, which consists of sets for objects and functions for morphisms. This functor sends each open set to the data attached to that set, and each inclusion to the corresponding restriction. Since sheaves are functors, maps between sheaves are maps between functors, i.e., natural transformations. Thus, sheaving pertains to a particular universal natural transformation, i.e., a second-order universal morphism.

The category theory concept of universal morphism is central to an explanation of systematicity (Phillips and Wilson, 2010). Conceptually, a universal morphism is the “best” possible construction (definition 15 in Appendix of the Supplementary Material). We have already seen two examples: product and constrained product. In general, the categorical product of two objects *A* and *B* is the best possible way of constructing an object that affords the recovery of *A* and *B*. In the category set, the product is the Cartesian product *A*×*B* together with two functions (projections) that retrieve the first and second elements from each pair (example 2 in Appendix of the Supplementary Material). In regard to tables, the Cartesian product is just all pairwise combinations of rows from each table, which are retrieved by the (relational) projection operations. Similarly, the constrained product is a universal construction: the best possible way of combining two tables so that they agree on overlapping columns, which is just the natural join. The product is a special case of the constrained product in that the agreement is automatic. In category theory, the constrained product (natural join) is an instance of the universal construction, called *pullback* (definition 12 in Appendix of the Supplementary Material).

The relevance of these concepts to sheaves and sheaving is two-fold. Firstly, a presheaf must satisfy the gluing (and the locality) condition to be a sheaf. From a category theory perspective, the gluing condition is given by products (pullbacks). Thus, to be a sheaf, a presheaf must satisfy a certain universality condition. Secondly, sheaving also pertains to a universal morphism in the context of a category of presheaves and presheaf morphisms. In this sense, sheaving obtains the best possible sheaf for the given presheaf. We have already explained that systematicity results from universal constructions (Phillips and Wilson, 2010). Thus, sheaving is a universal form of generalization. Since the construction returns a sheaf, which itself is a form of universal construction, sheaving pertains to a kind of second-order systematicity.

## 3. Going beyond the data: sheaving in cognition

The sheaf theory constructions just presented are applied to cognitive domains.

### 3.1. Cue-target learning: product

In this section, we show why generalization is afforded by sheaving for a task requiring participants to learn a set of cue-target mappings that is the product of two sets of cue-target mappings (Phillips et al., 2016). More details of the sheaf theory basis for generalization in this task are given in the “Cue-target (product) task: generalization as sheaving” section of the Appendix in Supplementary Material.

The task was to learn cue-target maps where the cues were pairs of characters and the targets were colored shapes, e.g., (G, K) ↦ (red, square), (G, P) ↦ (red, triangle), and so on. In the product condition, the map was the product of a map from characters to colors and a map from characters to shapes, e.g., G ↦ red and K ↦ square, etc. The motivation for this task was to test the hypothesis that systematicity, or failure to exhibit systematicity is due to a cost/benefit trade-off: for a small number of mappings participants were expected to learn the training set without the overhead of inducing the universal (product) construction and thereby not demonstrate generalization to novel pairs (testing set); for a larger number of mappings, where the demand on learning each pair separately becomes excessive, participants were expected to induce the product construction and thereby demonstrate generalization (systematicity). Experimental results supported these predictions (Phillips et al., 2016).

**Figure 3:**
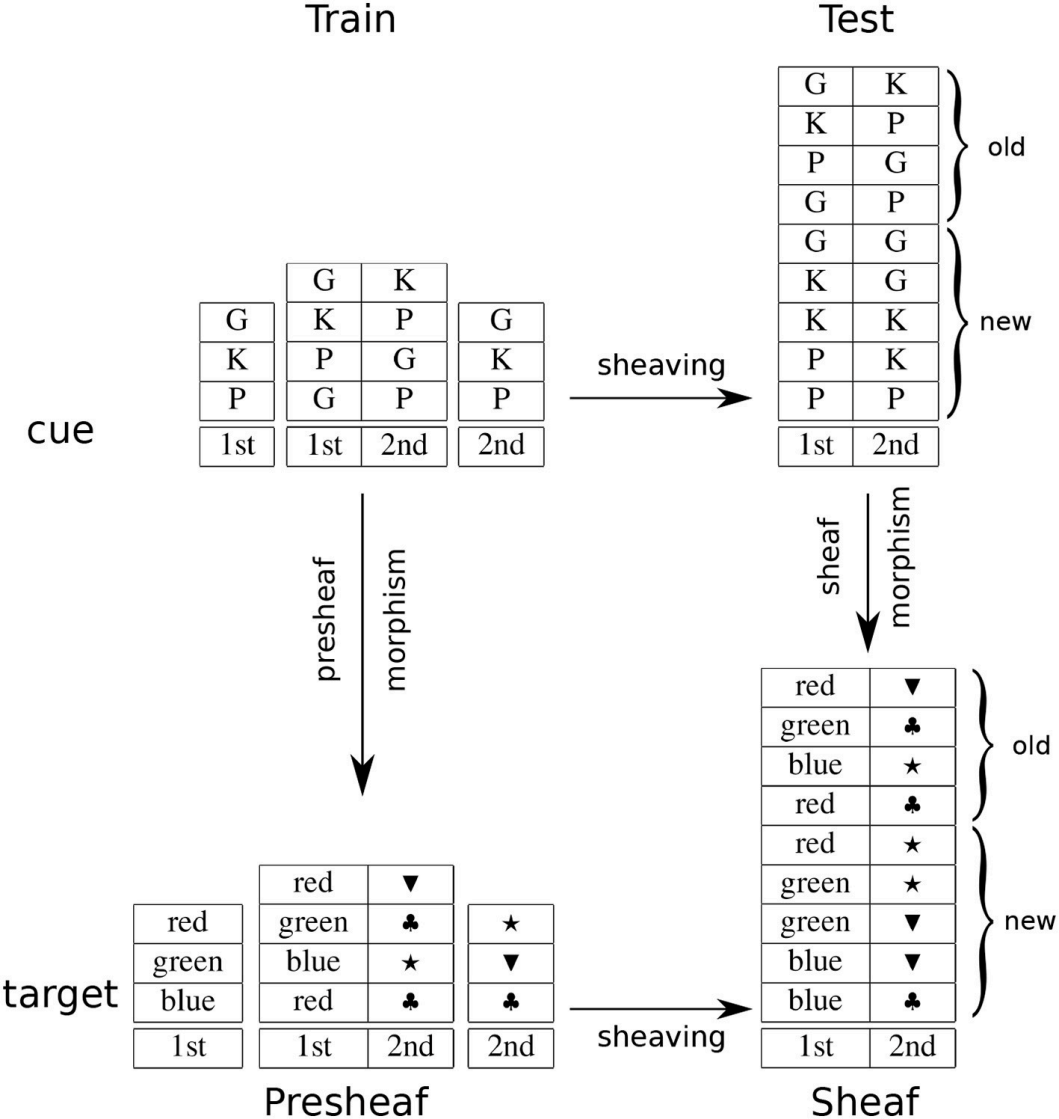
An example of generalization as sheaving.

The cue-target task investigated conditions that elicit universal constructions, hence systematicity. Here, we are interested in why such constructions are generated. According to the sheaf theory account, the relevant universal construction is a sheaf, which is obtained from another universal construction, sheaving. We have already shown how a product results from the sheaving process, in the previous section. Here, we show that the product map is a result of a sheaf morphism. From the relational database view of sheaves, a (pre)sheaf morphism is a map between relational databases. Sheaving as a basis for generalization is shown in Figure 3, where the sheaving constructions are the horizontal arrows, the presheaf morphism obtained from the training set is the left vertical arrow, and the sheaf morphism obtained from sheaving is the right vertical arrow, which affords generalization on the testing set. This arrangement is an instance of the commutative diagram (square) for a natural transformation (diagram 4 in Appendix of the Supplementary Material). Note that the morphism relating the test cues back to the training cues is given by the fact that sheaving involves an *adjoint functor* (see remark 16 in Appendix of the Supplementary Material). In psychological terms, participants recognize the importance of decomposing a pair of characters into their component characters: responses to novels pairs of characters can be determined by the responses to the individual characters as they appeared in other pairs during training.

The product task revealed that participants failed to exhibit systematicity for small maps even though the training set contained sufficient information to specify the underlying product and participants correctly learned the cue-target mappings for that set. From a sheaf theory perspective, this failure to demonstrate systematicity results from failure to identify the appropriate underlying topology. In this case, we regard the training set as a presheaf on an indiscrete topological space, in contrast to a discrete topological space. Recall, that an indiscrete topology on a set *X* consists of just the empty set and *X* as the open sets. A presheaf on an indiscrete topological space is trivially a sheaf: the gluing condition is automatically satisfied, because there is only one nonempty open set. Sheaving is just the identity transformation in this case, so no new rows are added to the table, hence participants do not go beyond the training data.

A psychological interpretation is that participants fail to recognize/represent the appropriate order relationship between the points of the space that correspond to the dimensions of the task, which impacts upon generalization. Recall (section 2.1) that a two-point space with the indiscrete topology corresponds to the preordered set: e.g., *A* ≤ *B* and *B* ≤ *A*, where *A* and *B* are the two points. The two points (dimensions) are equivalent, effectively regarded as a single point, hence sheaving has no effect in terms of generalization. By contrast, the discrete topology corresponds to the discrete ordered set: *A* ≤ *A* and *B* ≤ *B*, effectively regarding the two dimensions as independent, which affords generalization via sheaving. This difference can be interpreted as attentional load: spatial attention to stimuli as data attached to one vs. two locations.

Analysis of response data based on participant self-reports (Phillips et al., 2016) lends support to this interpretation. Upon completion of the experiment, participants were asked to report on how they performed the task. Participants were then divided into two groups indicating whether or not they were aware of the product structure of the mapping task. The aware group showed the same effects as observed in the original analysis. By contrast, the unaware group were not significantly above chance level performance in all conditions.

### 3.2. Visual search: constrained product

In a visual search task, participants are required to locate an object, designated as the target of search, in a display also containing nontargets. Typically, the target is uniquely identifiable by one or more features, e.g., color, shape, or orientation. The time to locate the target as a function of the number of objects in the search field is called the *search slope*. Search slope is typically shallower when targets can be identified by a single feature than when targets are identifiable by a conjunction of two or more features, e.g., color and shape (see Wolfe, 2003, for a review). Such behavioral differences led to the well-known and influential Feature Integration Theory of visual attention (Treisman and Gelade, 1980; Treisman and Sato, 1990), see Humphreys (2016) for a recent review. Although search slope may not be indicative of feature (shallow) vs. conjunctive (steep) search (Wolfe, 1998), recordings of monkey cortical activity support a feature vs. conjunction mode of attention (Buschman and Miller, 2007).

There is an obvious cost/benefit trade-off associated with having a features-detection system on one hand and a conjunctive construction system on the other. Dedicated feature units afford rapid response, but a unit is needed for each possible feature; (re)constructing conjunctions of features with dynamically reconfigurable units (i.e., units that can represent more than one conjunction) requires fewer units, but more time to detection. This trade-off is analogous to a trade-off in relational database design in that large tables are typically (re)constructed from smaller tables to save space, as well as to maintain data integrity (Halpin, 1995), but at the expense of longer query times. Construction of feature conjunctions is formally a natural join (Phillips et al., 2012, Text S3), hence it involves a sheaving process.

There is also a systematicity property in regard to feature binding: if one has the capacity to bind say features red and square, and features green and triangle, then one also has the capacity to bind features red and triangle, and green and square, regardless of whether one has seen that exact combination of features before. This property raises the familiar challenge of explaining why such a property exists: Why does having the capacity to bind, say, red with triangle and blue with square imply having the capacity to bind red with square and blue with triangle, assuming the capacity to recognize red, blue, triangle and square? The sheaf theory explanation is that systematicity of conjunctive features follows from a categorical universal construction, sheaving, which involves the natural join of feature maps, as explained in section 2.2.2. The universal morphism explanation for the systematicity of binding as a constrained product parallels the universal morphism explanation for cue-target pairs as products given in the previous section: the conditions for being a universal morphism (pullback) imply just those combinations.

Sheaving comports with the primacy of location-based feature maps (Riesenhuber and Poggio, 2004). In terms of the underlying topological space, the color and shape feature dimensions are closer to the location dimension than the color and shape dimensions are to each other. Accordingly, color-location and shape-location information are computed before color-shape-location information, which is typically expressed as faster response times (shallower search slopes) for feature than conjunction search (Treisman and Gelade, 1980; Wolfe, 2003).

The importance of the topology is reflected in the implications for binding. Dimensions are typically regarded as orthogonal and independent (as in the cue-target example of the previous section), which corresponds to a discrete topology. However, the discrete topology generates all possible conjunctions of features, not just those bindings present in the field of view.

Note that for ease of exposition, location is identified by a label/symbol for the example shown in Figure 2. However, location can also be modeled as a topological space, e.g., the product of topological spaces modeling the horizontal and vertical axes of two-dimensional display screen. Indeed, a parsimonious treatment of symbolic and spatial forms was one of the motivations for taking a sheaf theory approach, as foreshadowed in the Introduction.

In this example, we concerned ourselves with just the construction of representations for conjunctions of features, not with the process of searching for the target given those representations. A category theory approach to visual search has been discussed elsewhere (Phillips and Takeda, 2017). The theory employed there introduces another form of pullback, involving a change of base, that is beyond the scope of our current concern. Also, we have not considered the learning/development of conjunction search: e.g., young children are less efficient at conjunction search than older children and adults (Merrill and Lookadoo, 2004). Here and in the previous example, we concerned ourselves with representations and processes that pertain to a single topological space. More general situations that require changing the topological space are discussed in the next section.

## 4. Discussion

Our main purpose in this paper has been to (re)conceptualize generalization as sheaving: a process of “putting two and two together to make five,” so to speak. In the service of understanding cognition, sheaf theory appears to be a relatively unexplored area of mathematics—see, e.g., Goguen (1992) and Malcolm (2009) for applications to the related area of distributed systems, and Goguen (2018) for a discussion in regard to information integration. In this section, we discuss the prospects of a sheaf theory approach to learning and generalization, generally.

From a sheaf theory perspective, going beyond the data is about patching (or, gluing) local information to obtain new knowledge. The core property that affords sheaving is the ability to form the product of pieces of local knowledge constrained by their common source. So, by this account, sheaving should be evident in other cognitive abilities where products play a key role. Cognitive abilities such as matrix reasoning and transitive inference come to mind. A matrix reasoning task typically consists of a matrix of items (e.g., colored shapes) with the goal of identifying the item that goes in the empty cell location (e.g., *Raven's Progressive Matrices* Raven et al., 1998). For a relatively simple example, suppose that the rows are identified with colors: red, green and blue, and the columns are identified with shapes: circle, triangle and square, in those orders. The target that goes in the cell located at the third row and column is a blue square. This situation is similar to the cue-target learning task in section 3.1, as both involve a product of two dimensions. The topology consists of the two dimensions as open sets, and sheaving obtains the target by the product of the shape and color features attached to their respective dimensions.

More complex examples of matrix reasoning involve relations between the items within rows or columns. Models have been developed to account for simple and complex forms of matrix reasoning (Carpenter et al., 1990; Lovett et al., 2010). From our viewpoint, these situations involve data that have more internal structure than sets. The category theory approach to sheaves extends naturally to such cases as functors from a topological space to some other kind of category that has products, e.g., a category whose objects are groups, or rings (i.e., sets with one, or two internal operations). A challenge for the sheaf theory approach is to model both simple and complex forms of matrix reasoning.

Another cognitive ability pertaining to constrained products is *transitive inference*. Transitive inference has the form, if *A* is *R*-related to *B* and *B* is *R*-related to *C*, then *A* is *R*-related to *C*, where the relation *R* has the transitivity property. For example, if John is shorter than Mary and Mary is shorter than Tom, then John is shorter than Tom. In this situation, the premises are given by the order topology: *P* = {∅, {*P*_2_}, {*P*_1_, *P*_2_}} and *Q* = {∅, {*Q*_2_}, {*Q*_1_, *Q*_2_}}, where *P* and *Q* are the order topologies for the premises John is shorter than Mary and Mary is shorter than Tom, respectively. A capacity for transitive inference is regarded as crucially depending on an ability to integrate the premises into an ordered triple (Maybery et al., 1986; Andrews and Halford, 1998). In topological terms, integration corresponds to attaching data to a topology that encodes the three-term order, e.g., *T* = {∅, {*T*_3_}, {*T*_2_, *T*_3_}, {*T*_1_, *T*_2_, *T*_3_}}. Modeling this situation requires methods for changing the topological space. Here, category theory is again useful as there are two functors for changing the topology of a sheaf (presheaf): the *direct image functor* and the *inverse image functor* (Hartshorne, 1977; Mac Lane and Moerdijk, 1992). Another challenge, then is to model various aspects of transitive inference, including *pseudo-transitive inference* (Goodwin and Johnson-Laird, 2008), where the elements of the premises are locally, but not globally ordered.

A sheaf theory approach may also have something to say about the development of transitive inference and other reasoning tasks in terms of the development of the underlying topological space. Young children (below about 5 years of age) repeatedly have been shown to lack a capacity for transitive inference and a range of other reasoning tasks (Halford, 1984; Andrews and Halford, 1998, 2002). Some have argued that such capacities turn on the development of relational information processes (Halford et al., 1998, 2014; Penn et al., 2008), which has also been given a category theory account (Phillips et al., 2009). The category theory perspective attributed the difference to a capacity for products, including constrained products (pullbacks). We have already seen how these constructions are related to presheaves and sheaves, and the underlying topology. The sheaf theory approach presented here provides another related perspective on the development and evolution of intelligence, i.e., as a capacity to represent space. In particular, we noted that every set has two extreme topologies: indiscrete and discrete. For the collection of topological spaces on a given set, the indiscrete and discrete spaces are, respectively, the *coarsest* and *finest* topologies that can be given for that set, which are themselves instances of particular universal constructions. The relative coarseness/fineness of the underlying space alludes to developing progressively coarser/finer capacity to make spatial distinctions. For example, young children represent changes in shape differently than adults (Abecassis et al., 2001). The progression from holistic to category (class) based processes has been modeled computationally as learning/development via “intersection discovery” (Doumas and Hummel, 2010), by a symbolic connectionist model (DORA; Doumas et al., 2008). In our sheaf theory view, intersection discovery connotes development of a topological space.

The process of intersection discovery in DORA raises the possibility of developing a neural semantics for our category/sheaf theoretic approach to systematicity and generalization, as the pullback is a kind of intersection: in the category of sets and inclusions the pullback is just set intersection; in the category of sets and functions the pullback is the set of points that intersect (agree) on their images. DORA uses the role-filler binding method of the LISA model (Hummel and Holyoak, 2003) to induce relational representations via the interaction between proposition units representing relations, role-filler units representing the binding of values to relational roles, and feature units representing features of the related fillers (values)—role-filler units that coactivate the same feature units tend to be bound together by units representing a common relation. Conceptually, this arrangement is akin to a pullback of functions *f*:*A* → *C* and *g*:*B* → *C*, where the feature units correspond to the constraining object *C*, the interaction between role-filler and feature units to *f* and *g*, and the pullback object *A* ×_*C*_*B* to the units representing the relation. The dynamics of the DORA model are more complex than projections. So, the extent of a formal connection is not yet known. Developing a neural model for the theory would provide a basis for cost in terms of the neural resources needed to realize a universal construction.

Whether similar considerations apply to the development of conjunction search is a topic for future work. A capacity to represent conjunctions is just one aspect of visual attention, and there are multiple possible reasons for a change in search efficiency with age (see Merrill and Lookadoo, 2004, for a discussion). Here, we simply note that the pullback of two morphisms *f*:*A*→*C* and *g*:*B*→*C* is constrained by *C*. Thus, changing *C* (which means changing *f* and *g*) can change the number of elements constructed by the pullback, hence the number of elements selected for search, and thereby search efficiency.

Other potential applications are probability judgements that violate classical probability laws, e.g., *conjunction fallacy* (Tversky and Kahneman, 1983). In this situation, people judge the conjunction of two events *A* and *B* as more likely than either event *A* or event *B*: e.g., *P*(*A*∧*B*) ≥ *P*(*A*), which violates the classical probability law, *P*(*A*∧*B*) ≤ *P*(*A*). *Quantum probability theory* was introduced to explain such fallacies (see Busemeyer and Bruza, 2012, for an overview of theory and example applications). An important feature of this theory is contextuality where the act of measuring affects the outcome. The conditions for having quantum-like contextuality effects are closely related to the conditions for being a presheaf, but not a sheaf (Abramsky and Brandenburger, 2011). In these situations, the points of the topological space are measures and the values (data attached to the space) our outcomes, or outcome probabilities. The close connection between presheaves/sheaves and contextuality suggests that sheaf theory can also be applied to address contextuality effects in cognition.

Presheaves involve three kinds of morphisms, in addition to inclusions and restrictions: (1) morphisms from the topological space to the data, i.e., presheaves/sheaves, (2) morphisms between presheaves, i.e., presheaf morphisms, and (3) morphisms from sheaves on one topological space to sheaves on another topological space. We have primarily concerned ourselves with the second kind, in the form of sheaving, with regard to the generalization aspect of learning. However, for a more complete picture, we also need to consider how the first and third kinds of morphism pertain to other aspects of learning.

The first kind of morphism is important with regard to training and the partial state of knowledge acquisition. In particular, one difficulty with a category theory approach to cognition is how to model partial knowledge (Navarrete and Dartnell, 2017). With sheaf theory, partial knowledge can be related to the data attached to open sets and their restrictions. For example, the presheaf in Figure 2 has the empty set as the data attached to the open set, Feature, hence the associated restriction maps are empty maps. This situation reflects a temporary state of having partial (no) knowledge about, or representation of color-shape binding. In the context of learning, partial acquisition of knowledge can be modeled as a subset of the sections (rows) attached to an open set. How data get attached to open sets as a result of learning is a topic of future work.

The third kind of morphism is important in regard to explaining the transition from non-systematicity to systematicity. As mentioned in section 2.2.1, failure to generalize for small tasks can be attributed to having an indiscrete topological space. However, this account raises the question of why/how participants recognize the need to (re)represent space as a discrete topology to afford generalization. The cost/benefit hypothesis (Phillips et al., 2016) may help here, because the discrete topology (generally) contains more open sets than the indiscrete topology on the same set, hence requires more resources to represent. How cost/benefit interacts with learning in a sheaf theory setting is also a topic for further research.

Throughout this paper, we have focussed only on interpreting the gluing condition for a sheaf as a formal basis for the ability to go beyond the data. However, a presheaf must also satisfy the locality condition to be a sheaf. The locality condition says that gluing must be unique. Sheaving in this situation essentially identifies the alternatives as being the “same” data up to an equivalence. A detailed exposition will take us too far afield, however, this situation is like treating different instances of an object as the same object up to some equivalence relation. This situation typically does not arise in a relational database, because the relational database schema is essentially treated as a discrete topological space, in which case all rows must be unique, i.e., the locality condition is automatically satisfied. In a cognitive context, the locality condition may also have interpretations in terms of treating two distinct entities as the same thing: generalization on the basis of object class, rather than object instance.

Some authors have argued that the architecture of cognition (i.e., the basic processes and ways of combining such processes to afford cognition) is a “kludge” of disparate abilities that are somehow patched together to give the illusion of a well-organized system (Clark, 1987; Marcus, 2008). Be that as it may, viewing cognitive architecture as a hodgepodge of subsystems begs the question of why the system does actually work coherently, for the most part. The sheaf theory view presented here says that patching is a universal construction: an optimal solution to reconciling differences between subsystems put together as a kludge.

A sheaf can be likened to a kind of analogy in that the relations (inclusions) in the source domain (topological space) are mapped to relations (restrictions) in the target domain (data attached to the space), cf. *structure mapping theory* (Gentner, 1983). Category theory has been used as a basis for children's difficulty with understanding and exploiting the common relations in a reasoning problem (Halford and Wilson, 1980), and as an approach to analogy (Navarrete and Dartnell, 2017). However, a sheaf is a *contravariant* functor: the directions of arrows in the source are reversed in the target, which may strike some people as puzzling, given that analogy is typically conceptualized as a *covariant* mapping: the directions of arrows in the source and target are the same. One can conceptualize the role of contravariance in sheaf theory as persistence. Topological spaces can be built up by taking intersections and unions of the open sets in a basis set. Inclusions order open sets by size from small to large. A global property can be regarded as a property that *persists* over all the open sets—a property that is systematic as opposed to idiosyncratic (specific) to just some open sets—as we zoom in on smaller regions of space.

An important topic for further work is to explain how the cost/benefit proposal is supposed to interact with the construction of universal morphisms, as mentioned in the Introduction. An apparently straightforward approach would be to assign a cost to the alternative routes (see Phillips and Wilson, 2016b, for a discussion). However, this approach requires independent justification for the costs assigned, lest the cost/benefit principle becomes another *ad hoc* assumption, i.e., an assumption motivated solely to fit the data (Aizawa, 2003). Independent motivation may come in the form of empirical measures of the cost of each supposed alternative. For such purposes, a split-screen paradigm was developed to examine cost in the context of feature vs. conjunction visual search (Phillips et al., 2017). In this paradigm, participants could search for the target object in either the left or right visual field, which corresponded to feature or conjunction search. Search time when only one field was presented provided independent measures of the baseline costs of feature and conjunction search, which were then used to assess whether participants chose the alternative of least cost when both alternatives where presented at the same time. Analysis indicated that the choice of search field depended not only on the relative costs of the alternatives, but also on the cost of that assessment (Phillips et al., 2017). In this way, a categorical account of least cost may provide a principled explanation for the interaction between cost/benefit and universal construction, and its implications for systematicity and generalization.

Going beyond the data is a ubiquitous cognitive capacity in need of a theoretical explanation to motivate modeling as more than just an exercise in data fitting. The theoretical picture painted here is a view beyond local perception of the world. This sheaf theory approach formalizes our propensity to connect the dots. After all, that's what people do.

## Author contributions

The author confirms being the sole contributor of this work and approved it for publication.

### Conflict of interest statement

The author declares that the research was conducted in the absence of any commercial or financial relationships that could be construed as a potential conflict of interest.
