# Supplementary material for: Going Beyond the Data as the Patching (Sheaving) of Local Knowledge
Source: Front Psychol. 2018 Oct 9;9:1926. doi: 10.3389/fpsyg.2018.01926 (PMC6189483; doi:10.3389/fpsyg.2018.01926)
Supplement: Supplementary file 1 [file Data_Sheet_1.pdf]

Appendix

Sets

Definition 1 (Power set)**.** Let S be a set. The *power set* of S, denoted $\mathcal{P}(S)$, is the set of all subsets of S, i.e., $\mathcal{P}(S) = \{A | A \subseteq S\}$.

Definition 2 (Cartesian product)**.** Let A and B be sets. The *Cartesian product* of A and B, denoted $A \times B$, is the set of all pairwise combinations of elements from each set, i.e. $A \times B = \{(a, b) | a \in A, b \in B\}$, together with two functions $p_1 : A \times B \to A$ and $p_2 : A \times B \to B$, called *projections*, that send each pair of elements to their first and second components: $p_1 : (a, b) \mapsto a$ and $p_2 : (a, b) \mapsto b$.

Definition 3 (Graph)**.** Let $f : A \to B$ be a function from set A to set B. The *graph* of f is the set of ordered pairs $\Gamma(f) = \{(a, f(a) | a \in A\}$.

Definition 4 (Restriction)**.** Let $f : B \to C$ be a function and A a subset of B. The *restriction* of f to A is the function $f|_A : A \to C$ having the graph $\Gamma(f|_A) = \{(x, y) \in \Gamma(f) | x \in A\}$.

Remark 1. The function $f|_A$ is essentially f with its domain "narrowed" to A.

Definition 5 (Preimage)**.** Let $f : X \to Y$ be a function and B a subset of Y. The *preimage* of B, written $f^{-1}[B]$, is the set of elements in X that f maps to B, i.e. $f^{-1}[B] = \{x \in X | f(x) \in B\}$.

Definition 6 (Topological space)**.** A *topological space* (X, T) consists of a set X together with a collection T of subsets U of X, called the *open sets* of T, such that

- the empty set and X are open sets, i.e. $\emptyset, X \in T$,
- every arbitrary union of open sets is an open set, and
- every finite intersection of open sets is an open set.

The collection T is called the *topology* on X. The topology is also denoted T_X. The topological space is simply denoted X when the topology is understood.

Example 1 (Topological space)**.** The following are topological spaces.

1. A *discrete topological space* is a set X with the topology given by the power set of X, i.e. $(X, \mathcal{P}(X))$.
2. An *indiscrete topological space* is a set X with the topology that consists of just the empty set and X, i.e., $(X, \{\emptyset, X\})$.

Definition 7 (Continuous function)**.** Let (X, T_X) and (Y, T_Y) be topological spaces. A *continuous function* is a function $f : X \to Y$ such that for each open set $B \in T_Y$ the preimage $f^{-1}[B]$ is an open set in T_X.

Definition 8 (Cover, open cover)**.** Let I be an index set, and (X, T) a topological space. A *cover* of a set $U \subseteq X$ is a collection of sets $C = \{U_i\}_{i \in I}$ whose union contains U, i.e. $U \subseteq \bigcup_{i \in I} U_i$. If every set U_i in C is an open set of T, then C is called an *open cover*. The sets U_i are called *covering sets*.

Categories

Definition 9 (Category)**.** A *category* $\mathbf{C}$ consists of a collection of *objects*, $\mathcal{O}(\mathbf{C}) = \{A, B, \dots\}$, a collection of *morphisms*, $\mathcal{M}(\mathbf{C}) = \{f, g, \dots\}$—a morphism written in full as $f : A \to B$ indicates object A as the *domain* and object B as the *codomain* of f—including for each object $A \in \mathcal{O}(\mathbf{C})$ the *identity morphism* $1_A : A \to A$, and a *composition* operation, $\circ$, that sends each pair of *compatible* morphisms $f : A \to B$ and $g : B \to C$ (i.e. the codomain of f is the domain of g) to the *composite* morphism $g \circ f : A \to C$, that together satisfy the laws of:

- *identity*: $f \circ 1_A = f = 1_B \circ f$ for every $f \in \mathcal{M}(\mathbf{C})$, and
- *associativity*: $h \circ (g \circ f) = (h \circ g) \circ f$ for every triple of compatible morphisms $f, g, h \in \mathcal{M}(\mathbf{C})$.

Example 2 (Category)**.** The following are categories.

1. The category **Set** has sets for objects, functions for morphisms, and function composition as the composition operation. Identity functions are the identity morphisms.
2. The *opposite category* $\mathbf{C}^{\mathrm{op}}$ has the objects of $\mathbf{C}$ for objects, and for each morphism $f : A \to B$ in $\mathbf{C}$ the morphism $f : B \to A$, i.e. the morphisms of $\mathbf{C}^{\mathrm{op}}$ are just the morphisms of $\mathbf{C}$ in reverse direction.
3. The *Product category* $\mathbf{C} \times \mathbf{D}$ has $\{(A, B) | A \in \mathcal{O}(\mathbf{C}), B \in \mathcal{O}(\mathbf{D})\}$ for its collection of objects, $\{(f, g) | f \in \mathcal{M}(\mathbf{C}), g \in \mathcal{M}(\mathbf{D})\}$ for its collection of morphisms, and pointwise composition.
4. Every topological space (X, T) is a category, simply denoted X or T, whose objects are the open sets of T and morphisms are the inclusions $V \subseteq U$ for $V, U \in T$.

Definition 10 (Terminal object)**.** In a category $\mathbf{C}$, the *terminal object* is an object, denoted 1, such that for every object Z there exists a unique morphism $u : Z \to 1$, also written $! : Z \to 1$.

Example 3 (Singleton set)**.** In **Set**, the *terminal object* is any singleton set, $\{*\}$. For every set Z there is just one function $f : Z \to 1$, which is the *constant function*, i.e. $f : z \mapsto *$ for every element $z \in Z$.

Remark 2. Constructions that are required to satisfy certain equations are conveniently expressed in the form of *commutative diagrams*: pairs of paths starting at the same object and ending at the same object are equal, where at least one path consists of at least two (non-identity) morphisms.

Definition 11 (Product of objects)**.** In a category **C**, the *product of objects* A and B is an object P (also denoted $A \times B$) together with two morphisms $p_1 : P \to A$ and $p_2 : P \to B$, such that for every object Z and every pair of morphisms $z_1 : Z \to A$ and $z_2 : Z \to B$, there exists a unique morphism $u : Z \to P$ (also denoted $\langle z_1, z_2 \rangle$, since it is determined by z_1 and z_2), such that $z_1 = p_1 \circ u$ and $z_2 = p_2 \circ u$, as indicated by the following commutative diagram:

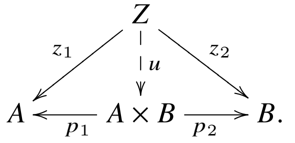

(1)

Example 4 (Cartesian product, set intersection)**.** The following are products of objects.

1. In **Set**, the product of sets A and B is the *Cartesian product*, $A \times B = \{(a, b) | a \in A, b \in B\}$ and the projections $\pi_1 : (a, b) \mapsto a$ and $\pi_2 : (a, b) \mapsto b$.
2. In the category of sets and inclusions, the product of sets A and B is set intersection, $A \cap B$, and the inclusions $A \cap B \subseteq A$ and $A \cap B \subseteq B$.

Example 5 (Product of functions)**.** Products of objects lead to the notion of a *product of morphisms*, which in **Set** is a *product of functions*. Suppose morphisms $f : A \to C$ and $g : B \to D$. The product of f and g is the morphism $f \times g : A \times B \to C \times D$, which is the *product of functions* $f \times g : (a, b) \mapsto (f(a), g(b))$, in **Set**.

Definition 12 (Pullback)**.** In a category **C**, the *pullback* of morphisms $f : A \to C$ and $g : B \to C$ is an object P (also denoted $A \times_C B$) together with two morphisms $p_1 : P \to A$ and $p_2 : P \to B$, such that for every object Z and every pair of morphisms $z_1 : Z \to A$ and $z_2 : Z \to B$, there exists a unique morphism $u : Z \to P$ (also denoted $\langle z_1, z_2 \rangle$, since it is determined by z_1 and z_2), such that the following diagram commutes:

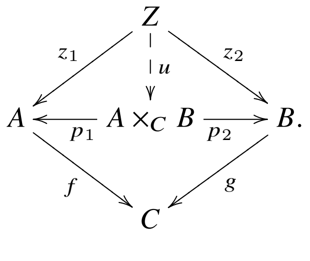

(2)

Example 6 (Natural join)**.** Let $R \subseteq A \times C$ and $S \subseteq C \times B$ be relations. The *natural join* of R and S is the set of triples $R \bowtie S = \{(a, c, b) | (a, c) \in R, (c, b) \in S\}$ obtained from the pullback indicated by the following commutative diagram:

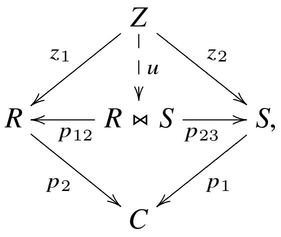

(3)

where p_{ij} is the projection of the ith and jth elements of each triple.

Remark 3. A product is equivalent to the pullback of morphisms $! : A \to 1$ and $! : B \to 1$, where $C = 1$ is the terminal object in $\mathbf{C}$. The terminal object provides no constraint on the product. In this case, the natural join reduces to the Cartesian product. Hence, a pullback is regarded a *constrained product*.

Definition 13 (Functor)**.** A *functor* is a "structure-preserving" map from a category $\mathbf{C}$ to a category $\mathbf{D}$, written $F : \mathbf{C} \to \mathbf{D}$, sending each object A and each morphism $f : A \to B$ in $\mathbf{C}$ to (respectively) the object $F(A)$ and the morphism $F(f) : F(A) \to F(B)$ in $\mathbf{D}$ such that:

- *identity*: $F(1_A) = 1_{F(A)}$ for every object $A \in \mathcal{O}(\mathbf{C})$, and
- *compositionality*: $F(g \circ_{\mathbf{C}} f) = F(g) \circ_{\mathbf{D}} F(f)$ for every pair of compatible morphisms $f, g \in \mathcal{M}(\mathbf{C})$.

Example 7 (Functors)**.** The following are functors.

- The *diagonal functor* sends each object and each morphism to their pairs, i.e. $\Delta : \mathbf{C} \to \mathbf{C} \times \mathbf{C}; A \mapsto (A, A), f \mapsto (f, f)$.
- The *product functor* sends each pair of objects and each pair of morphisms to their products, i.e. $\Pi : \mathbf{C} \times \mathbf{C} \to \mathbf{C}; (A, B) \mapsto A \times B, (f, g) \mapsto f \times g$.
- The *inclusion functor* sends each object and morphism in a category $\mathbf{C}$ as an object and a morphism in a category $\mathbf{D}$, i.e. $I : \mathbf{C} \to \mathbf{D}; A \mapsto A, f \mapsto f$.
- The *constant functor* $F_K : \mathbf{C} \to \mathbf{D}$ sends every object and morphism in $\mathbf{C}$ to the object K and identity morphism 1_K in $\mathbf{D}$. Constant functors are simply denoted by the target object, i.e. $K : \mathbf{C} \to \mathbf{D}$.

Remark 4. A functor $F : \mathbf{C} \to \mathbf{D}$ is also called a *covariant* functor. A functor $F : \mathbf{C}^{\text{op}} \to \mathbf{D}$ is called a *contravariant* functor.

Definition 14 (Natural transformation)**.** A *natural transformation* from a functor $F : \mathbf{C} \to \mathbf{D}$ to a functor $G : \mathbf{C} \to \mathbf{D}$, written $\eta : F \dot{\to} G$, is a family $\{\eta_A : F(A) \to G(A) | A \in \mathcal{O}(\mathbf{C})\}$ of morphisms in $\mathbf{D}$ such that $G(f) \circ \eta_A = \eta_B \circ F(f)$ for every morphism $f : A \to B$ in $\mathbf{C}$, as indicated by the following commutative diagram:

$$\begin{array}{ccc} F(A) & \xrightarrow{\quad \eta_A \quad} & G(A) \\ {\scriptstyle F(f)}\big\downarrow & & \big\downarrow{\scriptstyle G(f)} \\ F(B) & \xrightarrow[\quad \eta_B \quad]{} & G(B). \end{array} \tag{4}$$

Remark 5. A geometrical relationship between categories, functors and natural transformations is shown by the following, so-called *cell diagram*:

$$\mathbf{C} \overset{F}{\underset{G}{\rightrightarrows}} \mathbf{D}. \quad (\Downarrow \eta) \tag{5}$$

A category is a 0-cell (point), a functor is a 1-cell (line), and a natural transformation is a 2-cell (sheet). Hence, natural transformations are to functors as second-order functions are to first-order functions.

Remark 6. The collection of functors (objects) from the category $\mathbf{C}$ to the category $\mathbf{D}$ and the collection of natural transformations (morphisms) between those functors forms a *functor category*, denoted $\mathbf{D}^{\mathbf{C}}$.

Definition 15 (Universal morphism)**.** Let $G : \mathbf{D} \to \mathbf{C}$ be a functor and X an object in $\mathbf{C}$. A *universal morphism* from X to G is a pair (A, ϕ) consisting of an object A in $\mathbf{D}$ and a morphism $\phi : X \to G(A)$ in $\mathbf{C}$ such that for every object Y in $\mathbf{D}$ and every morphism $f : X \to G(Y)$ in $\mathbf{C}$ there exists a unique morphism $u : A \to Y$ such that $f = G(u) \circ \phi$, as indicated by the following commutative diagram:

$$\begin{array}{ccccc} X & \xrightarrow{\ \phi\ } & G(A) & \qquad & A \\ & {\scriptstyle f}\searrow & \big\downarrow{\scriptstyle G(u)} & & \big\downarrow{\scriptstyle u} \\ & & G(Y) & & Y. \end{array} \tag{6}$$

Remark 7. The *dual* version of universal morphism is defined by "reversing" the directions of the morphisms in the original definition: a universal morphism from a functor $F : \mathbf{C} \to \mathbf{D}$ to an object Y in $\mathbf{D}$ is a pair (B, ψ), as indicated in the following commutative diagram:

$$\begin{array}{ccccc} X & \qquad & F(X) & & \\ {\scriptstyle u}\big\downarrow & & {\scriptstyle F(u)}\big\downarrow & \searrow{\scriptstyle g} & \\ B & & F(B) & \xrightarrow[\ \psi\ ]{} & Y, \end{array} \tag{7}$$

i.e. for every object X and morphism g there exists a unique morphism u making the diagram commute.

Example 8 (Product as universal morphism)**.** The product of objects A and B is a universal morphism from the diagonal functor, Δ, to the object (A, B), i.e. the pair $(A \times B, (p_1, p_2))$—diagram 7 instantiates to

$$\begin{array}{ccccc} Z & & (Z, Z) & \xrightarrow{\quad (f,g) \quad} & \\ \Big\downarrow u & & \Big\downarrow (u,u) & & \searrow \\ A \times B & & (A \times B, A \times B) & \xrightarrow[(p_1,p_2)]{} & (A, B) \end{array} \tag{8}$$

where $u = \langle f, g \rangle$, which is equivalent to diagram 1. Terminal objects and pullbacks are likewise universal morphisms.

Remark 8. When universal morphisms exist there may be more than one, e.g., every singleton set is a terminal object in **Set**. However, universal morphisms are always *unique up to unique isomorphism* (i.e. there is only one such isomorphism that makes the diagram commute), and hence referred to as *the*, rather than *a* universal morphism.

Remark 9. Every universal morphism pertains to a natural transformation: the component ϕ of the universal morphism (A, ϕ) from X to $F : \mathbf{C} \to \mathbf{D}$ is the component $\phi_A : X \to F(A)$ of the natural transformation $\phi : X \dot{\to} F$ from the constant functor $X : \mathbf{C} \to \mathbf{D}$ to F. A natural join is a pullback and a pullback is a universal morphism. Hence, a natural join is *natural* in this technical sense.

Sheaves

Definition 16 (Presheaf)**.** Let (X, T) be a topological space. A *presheaf* is a (contravariant) functor $\mathcal{F}$ on X from T, considered as a category of open sets and inclusions, to the category **Set**, that is $\mathcal{F} : T^{\mathrm{op}} \to \mathbf{Set}$, such that for each open set U in T there is a set $\mathcal{F}(U)$ of elements, called the *sections* over U, and for each inclusion $V \subseteq U$ in T there is a morphism, called a *restriction morphism*, $f|_V : \mathcal{F}(U) \to \mathcal{F}(V)$ that satisfies the following laws:

- *identity*: for each open set U in T, the restriction morphism $f|_U : \mathcal{F}(U) \to \mathcal{F}(U)$ is the identity morphism $1_{\mathcal{F}(U)}$, and
- *compositionality*: for each triple of open sets U, V, W in T, if $W \subseteq V \subseteq U$, then $g|_W \circ f|_V = (g \circ f)|_W$.

If U is an open set of T, then $\mathcal{F}(U)$ is called the *sections* of $\mathcal{F}$ over U. Each element of $\mathcal{F}(U)$ is a called a *section*. A section over X is called a *global section*.

Definition 17 (Sheaf)**.** Let (X, T) be a topological space. A sheaf is a presheaf $\mathcal{F} : T^{\mathrm{op}} \to \mathbf{Set}$ that satisfies the following laws:

- *gluing* (existence): if $\{U_i\}_{i\in I}$ is an open cover of an open set $U \in T$, and if for each $i \in I$ a section $s_i \in \mathcal{F}(U_i)$ is given such that for each pair (U_i, U_j) of covering sets $s_i|_{U_i\cap U_j} = s_j|_{U_i\cap U_j}$, i.e. s_i and s_j agree on overlap, then there is a section $s \in \mathcal{F}(U)$ such that $s|_{U_i} = s_i$ for each i, and

- *locality* (uniqueness): if $\{U_i\}_{i\in I}$ is an open cover of an open set $U \in T$, and if $s, t \in F(U)$ such that $s|_{U_i} = t|_{U_i}$ for each U_i, then $s = t$.

Remark 10. The sheaf conditions are given by the following diagram:

$$\mathcal{F}(U) \xrightarrow{\langle \mathrm{res}_{U_i,U}\rangle_{i\in I}} \prod_{i\in I} \mathcal{F}(U_i) \underset{\mathrm{res}_{U_i\cap U_j,U_j}}{\overset{\mathrm{res}_{U_i\cap U_j,U_i}}{\rightrightarrows}} \prod_{i,j\in I} \mathcal{F}(U_i \cap U_j), \tag{9}$$

which is equivalent to the pullback of the restrictions $\mathcal{F}(U_i) \to \mathcal{F}(U_i \cap U_j)$ and $\mathcal{F}(U_j) \to \mathcal{F}(U_i \cap U_j)$.

Remark 11. In the case of the empty set, $\mathcal{F} : \emptyset \mapsto \{*\}$.

Remark 12. The collection of sheaves on a topological space (X, T) and morphisms between sheaves form a (functor) category, denoted $\mathbf{Sh}(X)$. Likewise, the collection of presheaves and presheaf morphisms on X is a category, denoted $\mathbf{Psh}(X)$.

Sheaving

Definition 18 (Sheaving)**.** Let (X, T) be a topological space, and $\mathbf{Psh}(X)$ the category of presheaves on X. *Sheaving*, also called *sheafification*, or *sheafifying*, is a map from a presheaf $\mathcal{F}$ in $\mathbf{Psh}(X)$ to the sheaf $\mathcal{F}^+$ (see remark 14), written $\theta_{\mathcal{F}} : \mathcal{F} \to \mathcal{F}^+$.

Remark 13. The family of maps $\{\theta_{\mathcal{F}}\}_{\mathcal{F}\in O(\mathbf{Psh}(X))}$ is the natural transformation $\theta : 1_{\mathbf{Psh}(X)} \dot{\to} I \circ Sh$, where $I : \mathbf{Sh}(X) \to \mathbf{Psh}(X)$ is the inclusion functor.

Remark 14. A procedure for sheaving and a proof that sheaving is universal (theorem 1), i.e. the pair $(\mathcal{F}^+, \theta_{\mathcal{F}})$ is a universal morphism, are given in (Hartshorne, 1977), Proposition-Definition 1.2, p. 64. The procedure consists of two steps that amount to collating the data at each point of each open set of the topological space (X, T):

1. determine the data attached to each point x of the open set U in T, called the *stalk* $\mathcal{F}_x$ at x, and

2. for each data element of the stalk join the data attached to the neighbourhoods of x, called the *germs*.

For tables, indicating presheaves on a space where each point is an open set, this procedure simply amounts to computing the projection at each point (column) x, and taking the product over all the projections (see example 9), hence the metaphor

of tying together stalks into sheaves. In general, points need not correspond to open sets, i.e. for a space (X, T) and a point $x \in X$ the set $\{x\}$ need not be an open set of T. In general, stalks and germs are determined by *(co)limits*, which are another kind of universal morphism (Hartshorne, 1977; Mac Lane & Moerdijk, 1992).

Example 9. A simple example illustrating the sheaving construction is given in figure 1.

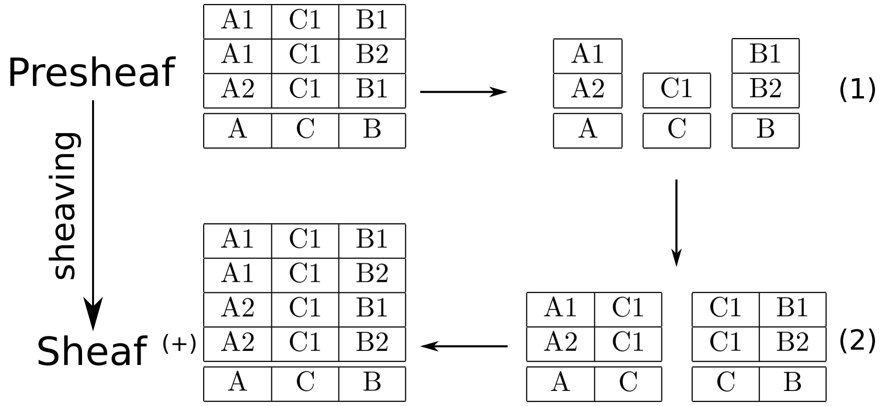

Figure 1: An example of the sheaving construction via (1) stalks and (2) tying together germs.

Theorem 1 (Sheaving)**.** Let $\mathcal{F} : X \to \mathbf{Set}$ be a presheaf and $\mathcal{F}^+$ the sheaf obtained by sheaving, i.e. $\theta_{\mathcal{F}} : \mathcal{F} \to \mathcal{F}^+$. The pair $(\mathcal{F}^+, \theta_{\mathcal{F}})$ is a universal morphism from $\mathcal{F}$ to the inclusion functor $I : \mathbf{Sh}(X) \to \mathbf{Psh}(X)$, as indicated by the following commutative diagram:

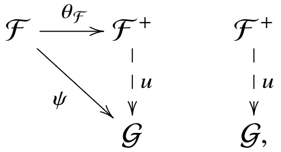

(10)

i.e. for every sheaf $\mathcal{G}$ and every presheaf morphism $\psi : \mathcal{F} \to \mathcal{G}$ there exists a unique sheaf morphism $u : \mathcal{F}^+ \to \mathcal{G}$ such that $\psi = u \circ \theta_{\mathcal{F}}$.

Remark 15. In short, sheaving is a universal morphism, or a universal construction.

Cue-target (product) task: generalization as sheaving

The cue-target product map is a product of a map from a set of characters to a set of colours and a map from the same set of characters to a set of shapes. The following data specifies an instance of the cue-target map for set size three: the set of characters, $Char = \{\mathrm{G}, \mathrm{K}, \mathrm{P}\}$, the set of colours, $Colour = \{\mathrm{red}, \mathrm{green}, \mathrm{blue}\}$, and the set of shapes, $Shape = \{\bigstar, \blacktriangledown, \clubsuit\}$. We have the following maps:

- $\mathit{char2colour} : \mathit{Char} \to \mathit{Colour}$; $\mathrm{G} \mapsto \mathrm{red}, \mathrm{K} \mapsto \mathrm{green}, \mathrm{P} \mapsto \mathrm{blue}$;

- $\mathit{char2shape} : \mathit{Char} \to \mathit{Shape}$; $\mathrm{G} \mapsto \star, \mathrm{K} \mapsto \blacktriangledown, \mathrm{P} \mapsto \clubsuit$; and

- $\mathit{char2colour} \times \mathit{char2shape} : \mathit{Char} \times \mathit{Char} \to \mathit{Colour} \times \mathit{Shape}$; $(\mathrm{G}, \mathrm{G}) \mapsto (\mathrm{red}, \star), (\mathrm{G}, \mathrm{K}) \mapsto (\mathrm{red}, \blacktriangledown), (\mathrm{G}, \mathrm{P}) \mapsto (\mathrm{red}, \clubsuit), (\mathrm{K}, \mathrm{G}) \mapsto (\mathrm{green}, \star), (\mathrm{K}, \mathrm{K}) \mapsto (\mathrm{green}, \blacktriangledown), \ldots$.

A subset of the set of cue-target pairs constitutes the training set. The testing set consists of all *old* and *new* cue-target pairs: pairs that appeared in the training set (old) and pairs that did not appear in the training set (new). We have the following data sets:

- training: $\{(\mathrm{G}, \mathrm{K}) \mapsto (\mathrm{red}, \blacktriangledown), (\mathrm{K}, \mathrm{P}) \mapsto (\mathrm{green}, \clubsuit), (\mathrm{P}, \mathrm{G}) \mapsto (\mathrm{blue}, \star), (\mathrm{G}, \mathrm{P}) \mapsto (\mathrm{red}, \clubsuit)\}$;

- old: $\{(\mathrm{G}, \mathrm{K}) \mapsto (\mathrm{red}, \blacktriangledown), (\mathrm{K}, \mathrm{P}) \mapsto (\mathrm{green}, \clubsuit), (\mathrm{P}, \mathrm{G}) \mapsto (\mathrm{blue}, \star), (\mathrm{G}, \mathrm{P}) \mapsto (\mathrm{red}, \clubsuit)\}$; and

- new: $\{(\mathrm{G}, \mathrm{G}) \mapsto (\mathrm{red}, \star), (\mathrm{K}, \mathrm{G}) \mapsto (\mathrm{green}, \star), (\mathrm{K}, \mathrm{K}) \mapsto (\mathrm{green}, \blacktriangledown), (\mathrm{P}, \mathrm{K}) \mapsto (\mathrm{blue}, \blacktriangledown), (\mathrm{P}, \mathrm{P}) \mapsto (\mathrm{blue}, \clubsuit)\}$.

Note that, in the training set, each character appears in each position, and each colour and shape appear in at least one of the targets.

The training and testing sets specify two presheaves and two sheaves, respectively. The cues and targets reside on two dimensions. Hence, the underlying topological space is (D, T), where $D = \{1\mathrm{st}, 2\mathrm{nd}\}$ is the set of dimensions and $T = \{\emptyset, \{1\mathrm{st}\}, \{2\mathrm{nd}\}, \{1\mathrm{st}, 2\mathrm{nd}\}\}$ is the discrete topology on D. Thus, we have:

- presheaves $\mathcal{F}_{\mathrm{cue}}, \mathcal{F}_{\mathrm{tar}} : D \to \mathbf{Set}$, and

- sheaves $\mathcal{F}^{+}_{\mathrm{cue}}, \mathcal{F}^{+}_{\mathrm{tar}} : D \to \mathbf{Set}$.

The sections of the presheaf $\mathcal{F}_{\mathrm{cue}}$ are specified by the rows of table 1. For instance, $\mathcal{F}_{\mathrm{cue}} : \{1\mathrm{st}, 2\mathrm{nd}\} \mapsto \{c_{\mathrm{gk}}, c_{\mathrm{kp}}, c_{\mathrm{pg}}, c_{\mathrm{gp}}\}$, where $c_{\mathrm{gk}} : 1\mathrm{st} \mapsto \mathrm{G}, 2\mathrm{nd} \mapsto \mathrm{K}$, etc.

c_{gk}	G	K
c_{kp}	K	P
c_{pg}	P	G
c_{gp}	G	P
	1st	2nd

Table 1: The presheaf $\mathcal{F}_{\mathrm{cue}}$.

The presheaf $\mathcal{F}_{\mathrm{tar}}$ is specified by the rows of table 2. For instance, $\mathcal{F}_{\mathrm{tar}} : \{1\mathrm{st}, 2\mathrm{nd}\} \mapsto \{t_{\mathrm{rb}}, t_{\mathrm{gc}}, t_{\mathrm{bs}}, t_{\mathrm{rc}}\}$, where $c_{\mathrm{rb}} : 1\mathrm{st} \mapsto \mathrm{red}, 2\mathrm{nd} \mapsto \blacktriangledown$, etc. The restrictions are given by projections onto table columns. For instance, for the inclusion $\{1\mathrm{st}\} \subseteq \{1\mathrm{st}, 2\mathrm{nd}\}$, we have the restriction morphism $c : c_{\mathrm{gk}} \mapsto c_{\mathrm{gk}}|_{1\mathrm{st}}, \ldots,$

t_{rb}	red	▼
t_{gc}	green	♣
t_{bs}	blue	★
t_{rc}	red	♣
	colour	shape

Table 2: The presheaf $\mathcal{F}_{\mathrm{tar}}$.

where $c_{\mathrm{gk}}|_{\mathrm{1st}} : \mathrm{1st} \mapsto \mathrm{G}$ is the section (function) c_{gk} restricted to the first column (dimension), etc. The sheaves, $\mathcal{F}^+_{\mathrm{cue}}, \mathcal{F}^+_{\mathrm{tar}}$ are likewise specified.

The collections of presheaves and sheaves on D form the categories $\mathbf{Psh}(D)$ and $\mathbf{Sh}(D)$, respectively. Hence, we have:

- the sheaving functor, $Sh : \mathbf{Psh}(D) \to \mathbf{Sh}(D) : \mathcal{F}_{\mathrm{cue}} \mapsto \mathcal{F}^+_{\mathrm{cue}}, \mathcal{F}_{\mathrm{tar}} \mapsto \mathcal{F}^+_{\mathrm{tar}}$,
- the inclusion functor, $I : \mathbf{Sh}(D) \to \mathbf{Psh}(D); \mathcal{F}^+_{\mathrm{cue}} \mapsto \mathcal{F}^+_{\mathrm{cue}}, \mathcal{F}^+_{\mathrm{tar}} \mapsto \mathcal{F}^+_{\mathrm{tar}}$, and
- the natural transformation, $sh : 1_{\mathbf{Psh}(D)} \dot{\to} I \circ Sh$,

which is indicated by the following commutative diagram:

$$\begin{array}{ccc} \mathcal{F}_{\mathrm{cue}}(D) & \xrightarrow{sh_{cue}} & \mathcal{F}^+_{\mathrm{cue}}(D) \\ {\scriptstyle train}\downarrow & & \downarrow{\scriptstyle test} \\ \mathcal{F}_{\mathrm{tar}}(D) & \xrightarrow[sh_{tar}]{} & \mathcal{F}^+_{\mathrm{tar}}(D). \end{array} \tag{11}$$

The map $train$ is the presheaf morphism obtained from training. The map $test$ is obtained from $train$ via application of the sheaving functor, and is related to $train$ by the natural transformation sh.

Diagram 11 shows the equational relationship between $test$ and $train$, i.e. $test \circ sh_{cue} = sh_{tar} \circ train$. The diagram says that traversal from the top-left to bottom-right corner is accomplished in either anticlockwise or clockwise direction. Thus, correct responses to novel (new) test cues can be computed in terms of the training set (see remark 16): response to a novel pair of cues is computed in terms of the responses to the individual cues learned during training. The $test$ morphism is related to the $train$ morphism by universal morphisms sh_{cue} and sh_{tar}. Hence, we have a kind of systematicity of generalization.

Remark 16. The morphism that relates the sheaf $\mathcal{F}^+_{\mathrm{cue}}(D)$ back to the presheaf $\mathcal{F}_{\mathrm{cue}}(D)$, which is not given by diagram 11, is given by the following facts:

- Sh and I constitute a pair of *adjoint functors* (Mac Lane & Moerdijk, 1992),
- every pair of adjoint functors induces a *natural isomorphism* (bidirectional natural transformation) (Mac Lane, 1998),

- hence, there is a commutative square with bidirectional horizontal edges such that *test* is given in terms of *train* via the composition of three other morphisms.

A detailed example is given in (Phillips, 2018).

The systematicity challenge and category/sheaf theory approach

The systematicity challenge for a theory of cognitive architecture (i.e. the putative basic cognitive representations and processes, and the ways these basic representations and processes combine to yield cognition) is to explain *why*, not just how, certain cognitive capacities always appear together. A typical example is the capacity to understand the phrase *John loves Mary* and the phrase *Mary loves John*. The theoretical challenge is to explain why these two capacities always appear together, or put the other way, why the situation of being able to understand one capacity but not the other does not occur (Fodor & Pylyshyn, 1988). In general, systematicity pertains to equivalence classes of structurally-related capacities (McLaughlin, 2009).

The classical and (revised) connectionist approaches assume some form of compositionality that covers the requisite equivalence classes. To wit, the classical approach assumes symbolic compositionality, i.e. there are symbols representing constituents and a symbolic process for combining those symbols to represent their complex host. Classical compositionality is supposed to explain systematicity because having these components to represent/process automatically extends to other combinations. Importantly, the common process for combining symbols is supposed to specify the equivalence class of cognitive capacities. Thus, one has either both or none of the capacities, but nothing in between. A similar explanation applies to connectionist approaches, sometimes called functional compositionality, where the mode of compositionality is supposed to be more generally some function, supporting the equivalence class of cognitive capacities, that is not necessarily juxtapositioning of symbols. In this case, the common function, which may be realized via common weighted connections in a neural network, specifies the requisite equivalence class of capacities.

Although both classical and connectionist accounts can be made to be consistent with systematicity, in and of themselves they are not sufficient. The basic problem is that the core assumption of shared symbols or functions (weighted connections) also admits situations in between, i.e. where some but not all of the requisite capacities are supported. So, some auxiliary assumption is required to pick out just the equivalence class. However, such auxiliary assumptions are *ad hoc* when their only purpose is to make the theory consistent with the property, rather than derive that property from the core principles and assumptions of the theory (Aizawa, 2003).

The category theory approach derives systematicity from universal morphism. In some respects, this approach is analogous to classical and connectionist compositionality. There are morphisms representing constituents and a composition operation for combining those morphisms into morphisms representing their complex hosts. So, systematically related capacities share a common (universal) morphism. However, the essential difference is that the common morphism is the universal morphism, not any shared morphism. So, one either has all or none of the members of an equivalence class of capacities, but nothing in between (Phillips & Wilson, 2010, 2016).

A universal morphism is also an "optimal" construction, in the sense that every other construction factors through it in a unique way. Conceptually (and formally), one can think of the universal morphism as a special node that is connected to every other node in a directed graph whose edges are the relations to other constructions. Consequently, every directed path from every node in the graph ends at the node corresponding to the universal morphism. A (*corecursive*) procedure was given to derive the universal morphism, in the general case (Phillips & Wilson, 2016). So, one can regard the process of constructing a universal morphism as a kind of optimization process.

Second-order systematicity and sheaving

Sheaving can be regarded as a kind of optimization process that is itself a universal morphism, hence the connection to second-order systematicity and learning. Graphically, since nodes correspond to processes, edges correspond to second-order processes, i.e. processes that modify (take/return) other processes, such as learning or development. Sheaving takes a presheaf (first-order process) and returns a sheaf (optimal first-order process). All other second-order processes factor through the sheaving process. Thus, sheaving is a natural (categorical) approach to second-order systematicity.

The sheaf theory approach to the cue-target task means that cue-target maps are second-order processes (presheaf/sheaf morphisms), because the cues and targets are first-order constructions: maps (functors) from a topological space to sets. By contrast, a typical functional approach is to treat cue-target maps as (first-order) functions between sets of cue/target elements. Consequently, the training and testing maps are related by sheaving morphisms, as indicated by diagram 11.

The importance of sheaving to second-order systematicity and generalization parallels the importance of the categorical approach to first-order systematicity: the capacity for generalization to novel (test) stimuli in one task instance implies the capacity for generalization to novel stimuli in another task instance (of the same structure) because both capacities factor through the same sheaving process.

References

Aizawa, K. (2003). *The systematicity arguments*. New York: Kluwer Academic.

Fodor, J. A., & Pylyshyn, Z. W. (1988). Connectionism and cognitive architecture: A critical analysis. *Cognition*, *28*(1–2), 3–71.

Hartshorne, R. (1977). *Algebraic geometry* (Vol. 52). New York, NY: Springer-Verlag.

Mac Lane, S. (1998). *Categories for the working mathematician* (2nd ed.). New York, NY: Springer.

Mac Lane, S., & Moerdijk, I. (1992). *Sheaves in geometry and logic: A first introduction to topos theory*. New York, NY: Springer.

McLaughlin, B. P. (2009). Systematicity redux. *Synthese*, *170*, 251–274.

Phillips, S. (2018). What underlies dual-process cognition? adjoint and representable functors. In C. Kalish, M. Rau, J. Zhu, & T. T. Rogers (Eds.), *Proceedings of the 40th Annual Conference of the Cognitive Science Society* (pp. 2250–2255). Austin, TX: Cognitive Science Society.

Phillips, S., & Wilson, W. H. (2010). Categorial compositionality: A category theory explanation for the systematicity of human cognition. *PLoS Computational Biology*, *6*(7), e1000858.

Phillips, S., & Wilson, W. H. (2016). Systematicity and a categorical theory of cognitive architecture: universal construction in context. *Frontiers in Psychology*, *7*, 1139.
